# Anonymization: The imperfect science of using data while preserving privacy

**DOI:** 10.1126/sciadv.adn7053

**Published:** 2024-07-17

**Authors:** Andrea Gadotti, Luc Rocher, Florimond Houssiau, Ana-Maria Creţu, Yves-Alexandre de Montjoye

**Affiliations:** ^1^ Imperial College London, Exhibition Road, London SW7 2AZ, UK.; ^2^ University of Oxford, Wellington Square, Oxford OX1 2JD, UK.; ^3^ Alan Turing Institute, 96 Euston Road, London NW1 2DB, UK.; ^4^ EPFL, CH-1015 Lausanne, Switzerland.

## Abstract

Information about us, our actions, and our preferences is created at scale through surveys or scientific studies or as a result of our interaction with digital devices such as smartphones and fitness trackers. The ability to safely share and analyze such data is key for scientific and societal progress. Anonymization is considered by scientists and policy-makers as one of the main ways to share data while minimizing privacy risks. In this review, we offer a pragmatic perspective on the modern literature on privacy attacks and anonymization techniques. We discuss traditional de-identification techniques and their strong limitations in the age of big data. We then turn our attention to modern approaches to share anonymous aggregate data, such as data query systems, synthetic data, and differential privacy. We find that, although no perfect solution exists, applying modern techniques while auditing their guarantees against attacks is the best approach to safely use and share data today.

## Teaser

Safe anonymization and sharing of personal data will be achieved by combining formal privacy methods with red-teaming.

## INTRODUCTION

Large datasets can be incredibly useful for statistical and research purposes, including use cases in the public interest. For example, census data benefits a wide range of stakeholders, from central governments to local authorities, businesses, and academics (*1*). Unlike traditional types of data, such as data from surveys and clinical trials, modern behavioral data are often automatically generated as a byproduct of users’ interaction with technology. This type of data is increasingly used by companies, governments, and researchers for analysis and decision-making. For example, mobile phone data have been used to assess population displacement caused by earthquakes (*2*), while social media data have been used extensively to study misinformation and the impact of digital media on democracy (*3*). The potential of data to benefit society has led to calls for its broader access and sharing (*4*, *5*). On the other hand, the collection and sharing of personal data at scale could jeopardize the fundamental right to privacy, opening the way to concrete privacy harms to individuals and society, such as mass surveillance, increased risks of blackmail, harassment, or identity theft (*6*, *7*).

Anonymization is among the main approaches to find a balance between sharing data and protecting individuals’ privacy. Intuitively, a dataset that has successfully undergone anonymization results in anonymous information, i.e., information where no individual is identifiable. The idea is that anonymized information cannot be linked back to an individual, and hence cannot be used to harm their privacy. Most data analysis applications do not require identifying users and can be carried out using anonymous data. Because of the strong protection it brings, anonymized data typically fall outside the scope of data protection laws, such as the EU’s General Data Protection Regulation (GDPR) (*8*) and the California Consumer Privacy Act (CCPA) (*9*). In particular, this means that anonymized data can typically be stored indefinitely, used for any purpose, and shared with any third party—all without the need for consent of individuals whose records were used to produce the anonymized data. What constitutes anonymous data thus needs to be carefully defined to ensure that anonymization does not create a loophole to avoid data protection law.

From a technical perspective, the standard for anonymity can be interpreted as a requirement of resistance against privacy attacks. Anonymized data should be robust against attempts to (re-)identify individuals and learn something about them. Designing, simulating, and evaluating privacy attacks is an important part of the research on anonymization, and their legal relevance has been explicitly recognized by regulators (*10*) and legal scholars (*11*). The other dimension of research on anonymization is on the defense side, designing techniques that transform data to achieve a high degree of anonymity while preserving good utility, i.e., the suitability of the anonymized data for the intended statistical analysis when compared to the original data. To conduct accurate research and derive valid insights, the statistical properties of the anonymized data needed for the purposes it is supposed to enable should be as similar as possible to the ones of the original data. As we will discuss in this review, inherent to anonymization is a privacy-utility trade-off that requires careful balancing between the risk of re-identification and the required suitability and accuracy of the data. This trade-off always exists, but an inappropriate technique may lead to a very bad trade-off, while an appropriate one may achieve an excellent trade-off. The privacy-utility trade-off is a fundamental principle of anonymization that guides all research in the field, and as such, it will be the cornerstone of this review.

As we will argue, traditional record-level de-identification techniques typically do not provide a good privacy-utility trade-off to anonymize modern data. Aggregate data—in its various forms, including machine learning (ML) models and fully synthetic data—can offer a better trade-off, but they do not inherently protect against privacy attacks. Our review of the literature shows that the best approach to successfully balance privacy and utility in practice is to carefully combine formal methods and an empirical evaluation of robustness against attacks, taking into account the context where the data are used or shared.

### Scope and rationale

This review offers a modern perspective on the field of anonymization, focusing on the challenges that high-dimensional data pose for traditional anonymization approaches and on the opportunities that state-of-the-art paradigms and techniques can offer to tackle such challenges.

Anonymity is both a technical and a legal topic. On one hand, computer scientists design anonymization techniques and study anonymity guarantees by devising and testing identification attacks on supposedly anonymous data. On the other hand, data protection and privacy laws such as the GDPR or the CCPA define what constitutes anonymous data and what rules, if any, apply to anonymous (including anonymized) data. Such definitions are typically very general, and hence, they are usually interpreted by regulatory bodies and ultimately—in case of litigation—by the courts.

The legal definitions of “anonymous data”—as well as those of related concepts, such as “de-identification” and “pseudonymization”—thus vary, sometimes substantially (*12*, *13*). The GDPR (Recital 26) defines anonymous data as “information which does not relate to an identified or identifiable natural person.” On the other hand, the CCPA and other US privacy legislations do not refer to anonymization and instead use the term “de-identification,” with each state using slightly different definitions (*14*). De-identification in the CCPA (and other US privacy laws) plays a role similar to anonymization in the GDPR—strongly reducing the legal obligations that apply to the processing of personal data.

The research community has been similarly debating what constitutes properly anonymous data from a technical standpoint. The conversation mostly revolves around which adversaries (i.e., persons attempting identification attacks) should be considered realistic—today and in the future—and how to determine and protect against the risks they may pose to privacy. Accurately describing the different views and how they diverge is outside the scope of this paper, and we refer the interested reader to Rubinstein and Hartzog (*15*), Elliot and Domingo-Ferrer (*16*), and Nissim and Wood (*17*) for a comprehensive account. To give a sense of the different perspectives, we mention the distinction drawn by Rubinstein and Hartzog, who speak about a debate between “formalists” and “pragmatists.” Omitting the many (and important) nuances of the debate, we can say that formalists prefer rigorous definitions of privacy and adversary models that are general and mostly independent of the context, while pragmatists believe that anonymization can be effective only by taking into account the situation where the data are released and relying on various mitigations strategies (e.g., legal contracts prohibiting re-identification, access control, and other security measures). Both communities can be critical of regulatory guidance, unsurprisingly in opposite ways (*18*, *19*).

The different approaches have also resulted in the development of different terminology and definitions. For example, the widely used concept of “quasi-identifier” (see the “Record-Level Data” section) is not defined consistently in the literature (*20*). Similarly, “record linkage” does not have a standard formal definition, with authors instead often providing ad hoc definitions tailored on specific types of data and anonymization techniques (*17*, *21*).

Here, we do not aim at presenting the many different technical definitions and interpretations, much less at reconciling them. We use definitions that are precise enough to describe the most important results accurately and clearly, but not so strict as to lose the nuances that often come with their meaning and use. In particular, we use “anonymization” to describe any processing of personal data that results in anonymous information, while we use “de-identification” to refer to a specific set of anonymization techniques (see the “Record-Level Data” section). Moreover, we focus mainly on the works published within the field of computer science since the late 1990s, although we occasionally mention articles outside this scope when necessary. This choice allows us to focus on current technical challenges and avenues for future research, as the past 20 years have seen a shift in the language and tools used to study anonymization (*7*, *22*, *23*).

In the ongoing debate between “formalists” and “pragmatists” and what constitutes properly anonymized data in practice, we strive to offer a balanced perspective that is informed by the GDPR concept of “means reasonably likely to be used” to identify a person. We discuss both the strengths and the weaknesses of different anonymization techniques, focusing especially on the challenges that arise when implementing them in practice. We believe such a contribution will be very useful for researchers who are already working on anonymization—but might be “pursuing rather different approaches to data confidentiality largely in isolation from one another” (*24*)—and for those who may want to start contributing to the field. We also hope for the review to provide a useful reference for policy-makers, regulators, and practitioners who work in the field of privacy and data protection.

### Summary of the review

In the “Background” section, we present some important concepts from the literature on anonymization. In the “Record-Level Data” section, we review the literature on anonymization when the goal is to release individual-level records, focusing on pseudonymization and record-level de-identification techniques such as *k*-anonymity. In the “Aggregate Data: Description and Types of Attacks” section, we then turn to aggregate data, describing the main use cases and types of attacks. In the “Attacks on Aggregate Data” section, we extensively review the literature on attacks against aggregate data in both the interactive and noninteractive settings, an active field of research. In the “A Theory for Releasing Aggregate Data: Differential Privacy” section, we present differential privacy, describing its properties, its strengths, and the challenges it poses for real-world applications. In the “Outlook” section, we summarize the review and provide our perspectives on future research directions.

## BACKGROUND

### Types of data

Anonymization is typically performed by a trusted party—the data curator—who has access to the original dataset that is used as an input for the anonymization process. The original dataset generally consists of individual records, where each record contains all data relating to the corresponding user. In the context of anonymization, it is useful to classify original datasets into three categories: structured, set-valued, and unstructured data (see Table 1). Set-valued data, unstructured data, and structured databases containing many columns are collectively referred to as high-dimensional data, as they can typically be represented as structured databases of high dimension, i.e., with many columns.

**Table 1.** Definitions and examples for the main categories of record-level datasets.

Concept	Description	Examples
Structured data	Datasets that are often represented as a table, where each row is a user’s record. A column contains each user’s value for the same attribute.	Survey, census, and health care cross-sectional data with demographic attributes (such as sex and age), observations (such as medical condition).
Set-valued (semistructured) data	Datasets where each user is associated with a collection of data points. Each data point typically contains information about “actions” or “events” relating to the user. The most prominent types of set-valued data are time series, where each point includes both the timestamp and the description of an action or event.	Most types of behavioral data, such as location data (containing individual trajectories, with time and location from GPS coordinates or cell towers, for each place visited), movies and videos watched online, and supermarket shopping data.
Unstructured data	Datasets that do not have a natural structured representation.	Text data (messages, tweets, letters), graphs (social networks), images and videos (face, body posture, fingerprint, iris).

Any dataset that contains individual records is referred to as record-level data (as well as individual-level data or microdata). In this review, we focus on the typical setting where the original datasets used as input contain record-level data. On the other hand, the output of an anonymization technique can take different forms. In some cases, the output can be record-level data as well. The most traditional type of anonymization, de-identification, produces record-level data and typically maintains a one-to-one mapping between the records in the original data and the records in the anonymized record-level data. De-identification has historically been the main approach, to the point that it is often just referred to as “anonymization.” To avoid confusion, we call it record-level de-identification, which will be the subject of the first half of this review.

Beside record-level data, the output of anonymization can take the form of, for example, summary statistics (such as mean values or marginal distributions), coefficients of a linear regression model, a trained generative neural network, or even a dataset of records sampled by such a neural network—what is often called synthetic data. With a possible abuse of language, we refer to all these types of data with the expression aggregate data, as they are obtained by aggregating information across individuals. We elaborate on this choice in the “Aggregate Data: Description and Types of Attacks” section. Perhaps contrary to expectations, aggregate data are not necessarily anonymous. Aggregate data will be the subject of the second half of this review.

### Privacy and utility

Anonymization is used to protect privacy while preserving utility. Privacy has many facets and forms, but in the literature on anonymization, it is mostly used as a synonym for confidentiality of an individual’s data. Other aspects of privacy are, for example, control and transparency over the use of one’s own information (*25*, *26*). The concept of privacy can also be extended from single individuals to groups (*27*). For simplicity, we follow the common practice for anonymization and use “privacy” to refer to confidentiality.

Similarly, there are many ways to quantify the statistical utility of anonymized data, such as statistical measures of error (e.g., distance metrics between distributions) or the difference in performance for data analysis tasks (e.g., classification tasks using ML) between the original and the anonymized data. These are widely used in research papers on anonymization to quantify the privacy-utility trade-off across different algorithms and parameters. In practice, utility is a more general notion that depends heavily on the purpose of the data analysis: The same anonymized data might be very accurate for one task and heavily inaccurate for another. We therefore use “utility” in a general, qualitative sense.

As we will discuss, when the input of anonymization is high-dimensional, using anonymization to release record-level data as output (preserving both privacy and utility) is generally hard, if not impossible. Most techniques for record-level de-identification have been designed for structured data with few attributes, which used to be the most common type of data before the advent of big data. These techniques mostly rely on the possibility to clearly define a limited number of attributes that may be known to an adversary (e.g., a person’s sex and age, but not their confidential answers to a survey). Such a distinction is often difficult or impossible to make for set-valued and unstructured data. For example, if a set-valued dataset contains the items purchased by a certain user, there might be no way to establish in advance which purchases may be known to an adversary. Similarly, in social network data, re-identification may be possible by exploiting the overall structure of the graph, which cannot be captured by a fixed set of attributes of the nodes.

### Trust model and adversary model

We focus on anonymization that considers a centralized trust model: The data curator acts as a central trusted entity with full access to the original (sensitive) records. The curator processes the dataset of records through an anonymization technique and shares the resulting data with one or more untrusted analysts. This is the model commonly assumed (sometimes implicitly) in the literature. The dual goal of anonymization is to preserve the statistical utility of the data for the analyst’s needs, while preventing a malicious analyst from performing a successful privacy attack, which would entail a privacy breach. A malicious analyst who attempts an attack is called adversary (attacker and intruder are also commonly used). When researchers propose a privacy attack, they rely on an adversary model. This is a description of the goals (i.e., the privacy breach the adversary is aiming at) and capabilities of the adversary, most notably the auxiliary information that is available to them. The adversary model typically has a target user (or victim), and the adversary’s goal is to infer some information about the user that would constitute a privacy breach. In attacks against record-level data, the auxiliary information typically consists of information about the target user. In attacks against aggregate data, auxiliary information often includes information about other users as well—for example, the adversary may be assumed to have access to part of the original dataset or an auxiliary dataset with a similar distribution. Ultimately, researchers propose attacks to identify vulnerabilities in anonymization mechanisms and propose new defenses or mitigations.

Other trust models are also considered in the computer science literature. The two main ones are the distributed model—used, e.g., in secure multiparty computation (MPC) (*28*) and federated learning (*29*)—and the local model—used, e.g., in local differential privacy (*30*).

## RECORD-LEVEL DATA

### Pseudonymization

Pseudonymization is the process of removing direct identifiers (such as name, phone number, email address, and national insurance number) or replacing them with a random pseudonym (*31*). We note that pseudonymization sometimes refers to a broader (and likely stricter) set of techniques (*32*), but in this review, we use the traditional definition. Pseudonymization can be typically used as a first step toward anonymization, but some practitioners have often—and incorrectly—considered it sufficient to obtain anonymous data, simply assuming that the absence of direct identifiers would prevent re-identification.

It has long been known that pseudonymized records are often vulnerable to a broad range of privacy attacks, called linkage attacks. These attacks aim to link a pseudonymized record to an identified record about the same user, which the adversary holds as auxiliary information. Proposed in 1959 by Newcombe *et al.* (*33*), linkage attacks were popularized on structured databases only in 1997 by Sweeney (*34*) who famously re-identified the governor of Massachusetts within a medical insurance dataset by linking his data with voter registration records. Sweeney showed that a person’s date of birth, sex, and postal code attributes contained in a public voter registration dataset were enough to link voter registration and medical records, allowing one to re-identify individuals and learn their medical information.

Linkage attacks can be divided into three classes of increasing complexity: exact matching attacks, robust matching attacks, and profiling attacks. Exact matching attacks refer to simple linkage attacks that match records using the exact same attribute values across two (or more) datasets—e.g., a target user’s date of birth, sex, and postal code in two different datasets. Robust linkage attacks aim to link two records even when they do not share the exact same attribute values, for example, due to inconsistencies or noise. Finally, profiling attacks aim to link records across datasets that refer to different nonoverlapping time periods such as two different years.

The basic property that makes all linkage attacks possible is uniqueness: If the dataset contains enough attributes, most records are inevitably unique in the database, meaning that no other record shares the same attributes. Hence, an adversary knowing a target user’s attributes as auxiliary information would be able to find the target user’s record.

Uniqueness is an inherent property of most high-dimensional datasets. Because the combinations of data values are many more than the number of records, these datasets are sparse: No two records look the same nor, often, similar. The amount of information collected nowadays about each individual means that modern datasets are often high-dimensional. While in 1790, the first US Census asked only four questions (*35*), modern data collection from governmental and private-sector organizations produces detailed individual profiles. For example, US governmental agencies maintain more than 2000 databases of personal data (*36*), while the data broker Acxiom claims to have about 1500 pieces of information per consumer (*37*). As we will now discuss, high-dimensional datasets are extremely vulnerable to linkage attacks.

Exact matching attacks have been demonstrated on structured data such as demographics data (*38*, *39*) and health data (*40*), set-valued data such as location (*41*, *42*) and credit card data (*43*), and unstructured data such as social network data (*44*, *45*), browsing histories (*46*), and genealogical trees (*47*). In particular, exact matching attacks on set-valued data can be carried out with very limited auxiliary information. To capture this property, de Montjoye *et al.* (*41*) proposed the unicity metric that measures the fraction of records in a set-valued dataset that are unique given a number of random points. Using this metric, they showed that four points are enough to uniquely identify 95% of individuals in a location dataset containing trajectories for 1.5 million people (*41*). Unicity has since been used to quantify the degree of identifiability of other types of high-dimensional data, such as datasets on shopping patterns (*43*), mobile app usage (*48*), and browsing histories (*46*). Some practitioners have raised questions on the relevance of reported values of unicity, as it is likely to vary with dataset size (*49*, *50*). Farzanehfar *et al.* (*51*), however, rebutted these claims by showing that unicity of location data decreases slowly with dataset size and remains high even in large-scale datasets.

Exact matching attacks are simple and effective, but rely on the crucial assumption that the adversary knows that the target user’s record is present in the dataset of pseudonymized records. Without this assumption, the adversary cannot confirm whether the (unique) matched record actually belongs to the target user or to someone else with the same characteristics. These considerations have also been used to motivate the use of subsampling—releasing only a fraction of records—as a defense measure to increase such uncertainty (*52*). This argument has been rebutted by Rocher *et al.* (*53*), who showed that the probability that a certain record is unique in the whole population—and therefore correctly identified in anonymous data—can be accurately estimated using only a small sample of records.

A more important limitation of exact matching attacks is that they assume the information present in the target dataset to be identical to the auxiliary information. Inconsistencies between datasets, due to incorrect or incomplete data, may sometimes prevent exact matching attacks. Robust matching attacks have thus been designed to address this limitation, for example, in location data (*54*), text data (*55*), and even social network graphs (*56*).

A general approach for robust matching was used by Fellegi and Sunter in 1969 (*57*). Their strategy consists in deriving a similarity metric d(*x*,*y*) between two records *x* in a dataset A and *y* in a dataset B to distinguish if these records are likely to originate from the same person (*x* = *y*) or not (*x* ≠ *y*). Given the complete distance matrix, several methods can be used to find a good (or even optimal) assignment between all the records in A and all the records in B: For example, the adversary can use a threshold-based rule [*x* = *y* if d(*x*,*y*) < σ] (*58*) or the Hungarian algorithm (*59*). A popular robust matching attack by Narayanan and Shmatikov (*60*), demonstrated on a Netflix dataset, is based on the same idea, but additionally incorporates some heuristics to improve robustness, such as giving more weight to rare attributes—which, intuitively, are more revealing of a user’s identity.

Although robust matching attacks can overcome inconsistencies across datasets, they nevertheless assume that the auxiliary information approximately matches the target dataset. By contrast, profiling attacks derive individual profiles that capture properties of each user’s behavior across time and contexts, and can thus be linked more robustly than raw records. For example, a profiling attack can be used to link trajectories in location datasets across different time periods. One possible way to do this is to extract points of interest (POIs) from location traces and then perform matching on these extracted features (*61*). Two typical POIs are the home location and the work location. On location data, profiling attacks have been proposed, which use, for example, histogram matching using likelihood-ratio tests (*62*) and frequency-based likelihoods (*54*, *63*) or Markov chain models (*59*, *64*–*66*) that capture regularity in the user behavior. Tournier and de Montjoye (*67*) recently improved on these results, proposing an entropy-based model that additionally estimates the correctness of the match. Profiling attacks have also been applied to interaction graphs derived from call detail records and Bluetooth close-proximity networks (*68*), smart meter recordings (*69*), and chess player actions (*70*). Modern deep learning techniques are likely to further increase the accuracy of attacks and the overall risk of re-identification (*68*). In particular, embedding allows profiling models to be trained on one dataset (e.g., one that is publicly available and thus accessible to any adversary) and then readily used to re-identify a person in a dataset from a similar distribution (*70*).

Linkage attacks are not just a theoretical risk: They have been proven effective on several real-world datasets, including publicly available ones. For example, individuals have been identified in publicly available health datasets in the state of Washington (*71*) and in Australia (*72*), and in a dataset about homicide victims in Chicago (*73*). In 2019, the *New York Times* re-identified the then-US President Trump’s tax records using supposedly anonymous public Internal Revenue Service (IRS) subsampled information on top earners (*74*). Practical linkage attacks on location data have been demonstrated several times on different datasets, such as on taxi rides (*75*), bike sharing trips (*76*), and GPS trajectories collected by apps such as Grindr (*77*). The latter case is also an example of the serious consequences that identification attacks can have on individuals: A member of the clergy allegedly resigned as a result of his identification in a Grindr dataset (*77*). Recently, the Federal Trade Commission initiated a lawsuit against the data broker Kochava over concerns for the sale of location data that could be used to identify women who visit abortion clinics (*78*).

Overall, policy-makers and regulators now acknowledge that pseudonymization is often not sufficient to obtain anonymous data. This is particularly the case in the EU, where the GDPR—which came into force in 2018—explicitly establishes that pseudonymous data relate to an identifiable individual and, hence, stay within the scope of data protection law. Despite this, pseudonymous data are still often considered—unknowingly or not—as anonymous by practitioners, and unfortunately, it is likely that many instances of identification by linkage in pseudonymous data are still happening today around the world.

### De-identification

In response to the re-identification risks of pseudonymized data, several techniques have been proposed over the past 50 years to anonymize record-level data. For decades, de-identification has been the main technical approach to anonymization, to the point that “de-identification” and “anonymization” are sometimes incorrectly used interchangeably. As mentioned in Introduction, the term “de-identification” is used with very different meanings, both technically and legally. In this review, we use “de-identification” to mean the set of techniques that modify the individual records in the original dataset to obtain a supposedly anonymous dataset. Hence, de-identified datasets are not aggregate data but rather contain individual-level records. Generally, de-identification would be expected to produce a dataset that allows general-purpose analytics, i.e., that preserves most statistical properties of the original dataset to a sufficient level of accuracy. In other words, it would be expected to provide a good privacy-utility trade-off for most statistical purposes, including those that are not known in advance.

Two main perspectives define the field of de-identification: The first one is based on heuristics, and the second one is based on privacy metrics. Although the techniques are mostly the same between the two perspectives, the approach they take is different.

The heuristics-based approach aims at applying a range of techniques to mitigate some linkage attacks. These techniques include generalization (e.g., replacing numerical attributes with intervals), randomizing attributes by adding noise or swapping them across records, suppressing outliers, and releasing only a sample of the records in the original dataset. A detailed review of these techniques, mostly studied within the statistical research community, is outside the scope of the review, but we refer the interested reader to the review by Matthews and Harel (*52*). While these operations might decrease the risk of re-identification, in many cases their impact is mostly analyzed from the perspective of the analyst and the impact on utility rather than privacy, and particularly on the properties—such as statistical bias—of commonly used statistical estimators [see, e.g., Kim (*79*) and Carlson and Salabasis (*80*)].

On the other hand, many privacy metrics have been proposed, many of which aim to quantify the risk that individuals can be identified in record-level data. A recent survey by Wagner and Eckhoff (*81*) describes over 80 privacy metrics and classifies them by the kind of property that they measure. It is beyond the scope of this review to discuss them in detail: To our knowledge, only a handful of these metrics have seen any adoption in practice, possibly also because it is often hard to determine what each metric means in terms of practical privacy guarantees (*82*, *83*). The privacy metrics that have attracted most attention are the ones relating to privacy models (also known as data protection models or confidentiality models). Inspired by the concept of security model in the field of cryptography (*26*), privacy models define the adversary model (the assumptions on the goal and capabilities of the adversary) and a set of requirements that the output dataset should satisfy to protect against that type of adversary (*84*, *85*). While a privacy model does not constitute an “absolute” guarantee of privacy, it is generally expected that the notion of privacy it captures is relevant and meaningful in practice.

The most well-known privacy model for de-identification is *k*-anonymity—proposed by Sweeney (*86*) in response to her own matching attack on the records of the governor of Massachusetts. It relies, like many models proposed later on, on the concept of quasi-identifier: an attribute that might be known to an adversary about the target user (e.g., the target user’s date of birth). *k*-anonymity requires that for each record in the dataset there are at least *k* − 1 other records sharing the same quasi-identifiers. This property is supposed to prevent record linkage, as no record is unique according to quasi-identifiers, which are assumed to be the only attributes known to an adversary. *k*-anonymity is typically achieved using a combination of attribute generalization (e.g., tree-based generalization), attribute or record suppression, and other techniques (*84*).

Privacy models based on quasi-identifiers have, however, been shown to suffer from serious limitations. First, it is very hard to reliably determine what auxiliary information might be available to an adversary, and hence which attributes should be selected as quasi-identifiers. If even just one more attribute is available to the adversary, the expected protections of the privacy model often collapse and the supposedly anonymous record may actually be heavily vulnerable to re-identification (*87*). Second, these privacy models do not generally address the common scenario of multiple releases: The combination of two *k*-anonymous datasets produced from the same original dataset may not be *k*-anonymous (*20*, *88*). In the language of differential privacy (see the “A Theory for Releasing Aggregate Data: Differential Privacy” section), this means that *k*-anonymity is not composable. Noncomposability poses serious challenges, as it is typically hard to produce a single anonymous dataset that achieves a good privacy-utility trade-off for all use cases. In most practical settings, the same original dataset will be used to produce multiple (supposedly) anonymous releases. Third, attacks have been proposed against supposedly robust privacy models. These include not only early attacks such as homogeneity and similarity attacks (*89*) but also more recent ones such as minimality attacks (*90*, *91*). Cohen (*20*) recently proposed a minimality attack against a real-world dataset where no record was unique, effectively proving that many privacy models (including *k*-anonymity) can be vulnerable even when applied in the most protective way (i.e., considering all attributes as quasi-identifiers).

The most serious challenge to de-identification in practice might, however, be on the utility side: For high-dimensional data, achieving meaningful anonymity guarantees while preserving good utility is very challenging, if not impossible (*84*). Intuitively, this can be seen as an instance of the curse of dimensionality: Since records in high-dimensional spaces are sparsely distributed, protecting against linkage attacks requires unacceptable levels of generalization or perturbation. This intuition has been formalized by Aggarwal in the context of *k*-anonymity in 2005 (*92*) and randomization in 2007 (*93*). In particular, Aggarwal analyzed the remaining utility after applying 2-anonymity—one of the weakest privacy models in terms of privacy protections. He showed that several measures of utility are severely affected when the dataset contains as few as 10 to 15 quasi-identifiers or more. These results, together with the empirical studies showing the vulnerability of high-dimensional pseudonymous data to linkage attacks, strongly suggest that it is generally hard to achieve a good privacy-utility trade-off with de-identification for high-dimensional data. Most researchers no longer consider de-identification a valid nor promising approach to anonymization in practice (*7*, *22*, *94*–*97*).

Despite the inherent limitations and demonstrated risks, de-identification remains popular in practice. Techniques such as *k*-anonymity are intuitively easy to understand and have the advantage of producing record-level data, which is what most data analysts are used to. However, the arbitrariness in the selection of quasi-identifiers makes it possible to claim privacy models such as *k*-anonymity even on large datasets, by assuming that only a few attributes qualify as quasi-identifiers. In contrast, modern approaches to anonymization tend to be more sophisticated, and their guarantees arguably less intuitive. On one hand, policy-makers and regulators have mostly acknowledged the risks of pseudonymous data, particularly in the EU, where the GDPR explicitly classifies pseudonymous data as data relating to an identifiable individual. On the other hand, to our knowledge, enforcement actions against the use of weak de-identification techniques have been limited so far, but some have taken place and are in the public domain. For example, the Federal Trade Commission in the US recently filed a lawsuit against Kochava for sale of sensitive location data, mentioning identifiability as an important risk factor (*78*). The result is an increasingly growing gap between the risk identified by anonymization research on one hand and anonymization policy and enforcement on the other hand.

## AGGREGATE DATA: DESCRIPTION AND TYPES OF ATTACKS

The vulnerability of record-level data to re-identification attacks stems primarily from the fact that information about single individuals is made available to analysts. Aggregating data across individuals in the form of, for example, summary statistics, count matrices, or ML models can help achieve a better balance between privacy and utility compared to record-level data. However, as we will discuss, this is not always sufficient: Aggregate data can still be vulnerable to privacy attacks. We note that the term “aggregation” is sometimes used to describe the generalization of attributes in record-level data (e.g., generalizing a postcode to the larger district). In this review, “aggregation” exclusively refers to the application of aggregating functions across records, not within records.

Aggregate data are typically released in either an interactive setting or a noninteractive setting. In the interactive setting, a data query system lets analysts request specific statistics by sending queries to an interface that supports a certain query language. Answers to the queries are computed on a dataset stored on a trusted remote server controlled by the data curator, allowing the analyst to choose and adapt new queries based on the results of previous ones. Before the results of the queries are returned to the analyst, both the queries and their results are modified or curated to protect privacy. Ideally, a data query system would enable general-purpose analytics on the underlying dataset, i.e., allow analysts to send many queries of different types, using a rich and flexible query language such as SQL, to perform sophisticated analyses easily and interactively (*98*). In a recent survey-based study, Garrido *et al.* (*99*) interviewed 24 privacy practitioners across nine companies and found that, to many of them, support for SQL is important when considering the adoption of tools for privacy-preserving data analysis.

In the noninteractive setting, the data curator chooses in advance which computations are performed on the original data and then releases the aggregated outputs to the analyst. An example of the noninteractive setting is the release of official statistics by national statistical agencies. The noninteractive setting also encompasses the practice of releasing or giving access to ML models trained on user data, e.g., ML models for text-to-speech, face recognition, sentiment analysis, and human-like text generation. These models are then released or made available through an application programming interface (API), a practice called machine learning as a service (MLaaS) (*100*). It is important to note that even if an API is used, MLaaS falls strictly within the noninteractive setting. Although analysts can query the model freely, these queries are computed against the pretrained model. Unlike the interactive model, these computations do not access the original data in any way. Finally, an increasingly popular method to produce statistically useful data is synthetic data generation (SDG), a class of mechanisms that generate “artificial” data from original data. The goal is to generate and share record-level data—allowing analysts to run analyses using the same algorithms and pipelines that they would use on real records—while protecting privacy. Similarly to MLaaS, synthetic data fall within the noninteractive setting, as analysts only have access to synthetic data—and sometimes to the model used to generate them—but never to the original data.

We use aggregate data to refer broadly to any type of data produced by aggregation, including summary statistics, ML models, synthetic data, and answers to data query systems. We note that, while commonly used, this is an informal expression: To our knowledge, “aggregate” and “aggregation” are never defined formally. The reconstruction attacks discussed later can be seen as indication that it is hard to give a formal definition of “aggregation,” which agrees with its common informal meaning: If all the original records can be reconstructed based only on counts, the properties that distinguish aggregate data from record-level data are intrinsically elusive. Nevertheless, as the rest of this review will show, the separation between record-level data and aggregate data is a useful perspective to think about anonymization in practice.

It is important to emphasize that, in general, releasing only aggregate data substantially reduces the vulnerability to attacks compared to record-level data in practice. Yet, research has shown that, in some cases, even aggregate data can leak information about single records. These privacy attacks can be classified into three different categories: membership inference, attribute inference, and reconstruction (see Table 2).

**Table 2.** Types of attacks on aggregate data.

Attack	Description	Example of harms
Membership inference attack (MIA), also called tracing attack	MIAs attempt to determine whether records of a target user are present in the dataset that was used to compute the aggregate data. MIAs can also serve as a first step for more powerful inference attacks, which often rely on the assumption that the target user is a member of the dataset. They are often used as a “benchmark” to show that some aggregate statistics leak individual-level information.	Presence in a dataset can be sensitive in itself. For instance, for a dataset on individuals with a stigmatized health disease, a successful membership attack could indicate that an individual has been diagnosed with this disease.
Attribute inference attack	These attacks aim at learning information that is contained in the original record-level dataset.	The adversary can learn the value of a sensitive attribute in the original data—e.g., political affiliation or medical condition—or characteristics that emerge from the overall data of the individual without being explicitly part of the data—e.g., personality traits.
Reconstruction attack	The adversary attempts to reconstruct some of or all the original record-level dataset using exclusively aggregate data, potentially without the need for auxiliary information.	Strictly speaking, reconstructions attacks are not re-identification attacks, but they allow the adversary to perform attacks on the original record-level dataset, such as linkage attacks (assuming that the reconstruction is sufficiently accurate).

## ATTACKS ON AGGREGATE DATA

Attacks differ considerably between the interactive and the noninteractive setting. In the interactive setting, the adversary can freely define the queries that are answered, and has therefore a lot of flexibility in defining which aggregate information is disclosed. This may allow the adversary to actively exploit vulnerabilities in the system (see example below). By contrast, in the noninteractive setting, the data that are disclosed are decided by the curator, and are therefore less likely to contain “ready-to-use” vulnerabilities that can be directly exploited. Attacks may still be possible, but these need to carefully leverage the cumulative leakage of individual-level information from the data—something that may involve more sophisticated methods and may often require large amounts of data being released or stronger assumptions on the data and on the auxiliary information available to the adversary.

In other words, data query systems present a larger attack surface: The adversary can craft very complex and often “unnatural” queries that would not typically be released in a noninteractive setting. These queries, in combination, may exploit a vulnerability in the system to extract individual-level information (performing membership or attribute inference) or even reconstruct the full dataset. The risk of vulnerabilities is particularly high in the case of general-purpose analytics. In this case, the data query system must necessarily support a rich query syntax (such as SQL, or at least a large subset of it) and allow the same analyst to issue many queries while preserving enough statistical utility. These requirements make the system harder to protect against attacks.

We illustrate the potential for attacks on aggregate data with two classic and simple examples. Suppose that a city administration has a dataset that includes, for every citizen, their date of birth, sex, postal code, and a binary attribute indicating whether they own any property in the city. The city administration sets up a data query system to allow independent researchers to analyze the data without sharing the original dataset with them. However, a person’s date of birth, sex, and postal code attributes are often sufficient to uniquely identify that person (see the “Record-Level Data” section). For example, a researcher could know a man (the target user) who was born on 1 January 1984 and lives in postal code 00187, and be confident that no other person shares the same attributes. If the system answers every query truthfully, a researcher could submit a query such as: How many persons are born on 1 January 1984, are male, live in postal code 00187, and own property? The answer can be either 1 or 0, which fully reveals whether the target user owns property or not, respectively.

Such an attack can be easily prevented by rejecting, for example, any query that selects less than five persons in the dataset. However, this protection can be easily circumvented by using two queries instead of one:

Q1: How many people own property?

Q2: How many persons own property and are not males who were born on 1 January 1984 and live in postal code 00187?

Neither Q1 nor Q2 would be rejected, because they both select a large number of people. However, because of the uniqueness of the target user’s attributes, the difference between the answers to Q1 and Q2 can again be either 1 or 0, depending on whether the target user owns property. This type of attack is called differencing attacks (or intersection attack) and is a very simple kind of inference attack on aggregate data.

Note that such simple attacks would generally not be possible in the noninteractive setting: In a typical scenario, it is very unlikely that a query like Q2 would be intentionally included in the summary statistics released by the curator. This is not the case for data query systems, which must generally allow for more flexibility. While several mitigations can be developed for these attacks, this is still an active area of research. While in principle, it would be possible to audit every query—taking into account all previously answered queries—to ensure that answers do not leak private information, Chin (*101*) shows that some query auditing problems are NP-hard and Kenthapadi *et al.* (*102*) show that rejecting a query can itself leak private information.

### Data query systems

Possibly because of the larger attack surface, the first principled study of attacks on aggregate data effectively considered the interactive setting. In 2003, Dinur and Nissim proposed the first linear reconstruction attack, which uses linear optimization to recover almost perfectly the full original dataset based only on noisy answers to queries (*103*). Notably, they show that the attack is effective on any system, as long as the adversary can issue specific types of queries that perform summation over arbitrary sets of users (defined by the analyst and typically chosen at random), and provided that the noisy answers are accurate enough on a large enough set of queries. The attack they propose requires a polynomial number of queries (in the size of the dataset). Linear reconstruction attacks have since been extended and improved by subsequent work, particularly to reduce the computational time and increase the robustness to noise (*104*–*107*). We note that, although Dinur and Nissim did not explicitly focus on the interactive setting, the type of queries used by the attack are unlikely to be released in a noninteractive setting. However, as we will discuss later, linear reconstruction attacks have since been extended to the noninteractive setting, including on summary statistics (*106*, *108*) and synthetic data (*109*). A survey by Dwork *et al.* (*110*) provides an in-depth overview of these subsequent results.

These results have led to the formulation by Dwork and Roth (*30*) of the Fundamental Law of Information Recovery, an informal principle stating that “overly accurate answers to too many questions will destroy privacy in a spectacular way”. Despite their generality in terms of robustness to different types of noise, the theoretical line of work on reconstruction attacks typically relies on the availability of queries that can freely select sets of users, for example, through user IDs. This is known as the row-naming problem (*111*, *112*). If the data query system does not support such queries, executing a reconstruction attack in practice may require additional assumptions or workarounds, such as specific vulnerabilities to exploit the available query syntax (*113*) or methods to select users based on their attributes (*114*, *115*).

Researchers have recently demonstrated that exploiting specific properties of the data query system can enable very strong attacks that are directly applicable in practice. For example, Matthews *et al.* (*116*) considered 35 US state-level web-based data query systems to share public-health relevant data, and showed that 9 of them were vulnerable to differencing attacks. Similar differencing attacks were proven effective against the platform deployed by Facebook for microtargeted advertising on their social network (*117*–*119*).

System-specific attacks have also been demonstrated on data query systems using more sophisticated privacy mechanisms. Asghar and Kaafar (*112*) proposed a denoising attack against TableBuilder, the system deployed by the Australian Bureau of Statistics to query census data. Their attack infers the exact (“denoised”) aggregate value that is supposed to be hidden by TableBuilder’s noise addition mechanism, which in turn can be used to mount attribute inference attacks such as differencing attacks.

Diffix, a commercial data query system claimed to provide “GDPR-level anonymization,” was proposed in 2017 and subsequently revised by Francis *et al.* (*120*, *121*). Diffix—whose specifications are publicly available—is likely the most sophisticated solution for privacy-preserving general-purpose analytics among those that do not enforce differential privacy. It uses a combination of privacy mechanisms, including multilayer pseudorandom noise, while enabling high-utility statistical purposes. Previous versions of Diffix were shown to be vulnerable to membership inference attacks (MIAs) (*122*), reconstruction attacks (*113*), and attribute inference attacks (*123*). In particular, Gadotti *et al.* (*123*) propose the first example of noise-exploitation attack on a deployed system, which exploits Diffix’s specific structure of the noise itself as a signal to infer sensitive user attributes and achieves high accuracy with a limited number of queries.

Cretu *et al.* (*124*) recently proposed an approach to automatically design attribute inference attacks, with the goal to enable data query systems to be tested at the “pressing of a button”. Their approach uses evolutionary search techniques to jointly optimize the set of queries to issue to the system and an ML classifier to learn to combine the answers to the queries to infer a victim’s sensitive attribute. They show that automated attacks can match or outperform manual attacks from the literature across a range of systems. Their algorithm, however, only supports a limited query language, and extending it to more complex query languages remains an open problem. Overall, these attacks show that thwarting attacks in the interactive setting is still a challenging open problem, and emphasize the need to test systems and consider mitigation strategies before deployment.

### Summary statistics

Aggregate data released in the noninteractive setting typically offer a narrower attack surface, as the data owner chooses what is released, and is thus generally “harder” to attack. We use “harder” in an informal sense to mean a combination of different factors, including the sophistication of the attack and the plausibility of the adversary model (particularly the amount and type of required information) on one hand and the amount and accuracy of the inferred information on the other hand. Since the adversary cannot freely define attack queries, the lack of this flexibility must typically be compensated either by a large amount of aggregate data being released or by other means—e.g., assuming that the adversary has access not only to auxiliary information about the target user but also to large amounts of information about other users. This is especially the case for predefined summary statistics, which are a common use case for noninteractive publication of aggregate data. These are often descriptive statistics that not only summarize important properties of the data, such as counts, mean values, or median values, but also include outputs of more complex analyses, such as experiment results from a scientific paper (e.g., correlation coefficients and test statistics).

In 2008, Homer *et al.* (*125*) published the first study of privacy attacks against commonly released summary statistics, demonstrating a MIA on a public genome-wide association study (GWAS). Their attack uses a statistical hypothesis test to infer membership using aggregate allele frequency statistics, a form of one-way marginals. Homer *et al.*’s results were later formalized, validated, and improved (*126*–*129*), with Backes *et al.* (*130*) extending the attack to microRNA data. In addition to the one-way marginals, Homer *et al.*’s attack and follow-up works rely on access to two types of auxiliary information: the individual-level record of the target user [specifically, a single-nucleotide polymorphism (SNP) profile] and the one-way marginals computed on a reference population. Intuitively, the attack exploits the fact that the target user’s record is likely to be a member if it is “closer enough” to the released marginals than to the ones computed on the reference population. The attack is made possible by the total information leaking from the large number of one-way marginals in high-dimensional genomic data. Inspired by these works, Dwork *et al.* (*131*) studied the potential of MIAs on one-way marginals from a general and theoretical standpoint, showing that releasing many marginals increases the vulnerability to the attack, while a higher number of users or larger noise negatively affect the attack. In particular, they proved that, for a fixed number of users and noise level, MIAs are always possible as long as a large enough number of marginals are released.

Following these studies on genomic data, MIAs have been studied in a range of other contexts. Buescher *et al.* (*132*) proposed an attack on smart meter data: Using a distance-based method similar to the one originally proposed by Homer *et al.*, they demonstrate how to infer membership in aggregate energy load profiles. On the basis of their results, Buescher *et al.* question previous claims from industry research that aggregating consumption measurements from only two households provides sufficient customer privacy. Pyrgelis *et al.* (*133*) proposed the first MIA against aggregate location data, and specifically density time series (i.e., hourly counts of how many people were at predefined locations). The attack uses auxiliary information about many users—a potentially strong assumption in the context of location data—to simulate many different aggregate samples with or without the target user, training an ML model to automatically distinguish between the two. Notably, their attack can work even when the auxiliary information and the aggregate data refer to different periods. Although the attack achieves very good accuracy on datasets of relatively small size, its effectiveness decreases with the size of the dataset (*133*, *134*). It is also unclear how the attack would perform on a real-world dataset, as Pyrgelis *et al.*’s evaluation selects the 10,000 most active users from an original dataset of 4 million users. Subsequent work by Pyrgelis *et al.* (*135*) found that combining several well-known privacy techniques such as sampling and perturbation can substantially reduce the attack’s effectiveness while preserving good utility. More recently, Voyez *et al.* (*136*) proposed another methodology for membership inference, based on linear programming. While their attack is very effective, it relies on a very strong adversary who has exact knowledge of all the records in the population (from which the records that form the original dataset are sampled).

Overall, there is substantial evidence showing that MIAs against summary statistics are possible in theory. However, most works in this area have limitations that should be taken into account when assessing the practical privacy risks. First, the effectiveness of these attacks can often decrease rapidly when the data are aggregated over more than a few thousand individuals. Second, the attacks often make very strong assumptions, especially on the adversary’s auxiliary information. In recent work, Bauer and Bindschaedler (*137*) investigated several MIAs in the literature under realistic settings on the type of aggregation, the auxiliary information, and the probability that a user is included in the dataset. They conclude that methodological assumptions in previous work might give a misleading picture of the risk, but emphasize that attacks can be effective on the most vulnerable users under realistic assumptions.

Contrary to membership inference, results on attribute inference and reconstruction attacks on summary statistics are more limited, with only a few attacks proposed so far. Wang *et al.* (*138*) design and evaluate an attribute inference attack against statistics commonly published in GWAS papers, such as *r^2^* correlation coefficients between SNPs. In two separate works, Kasiviswanathan *et al.* extend the linear reconstruction attack by Dinur and Nissim (*103*) to natural types of summary statistics such as *k*-way marginals (*108*) and estimators for linear and logistic regression (*106*). In their attack, the adversary is assumed to know the full individual-level records of all users in the dataset, except for one “sensitive” attribute that is unknown for all users. The adversary’s goal is to reconstruct the “sensitive” attribute for each user. While this allows the adversary to know which users are selected in each aggregate statistic (the row-naming problem mentioned previously), it constitutes an important limitation for the applicability of the attack in practice.

To the best of our knowledge, the only documented case of a successful linear reconstruction attack on real-world predefined summary statistics are the ones recently performed by the US Census (*139*, *140*). J. M. Abowd (US Census’s chief scientist) claimed that the Census Bureau was able to simulate a reconstruction attack using 6 billion of the over 150 billion statistics released in the 2010 census, which would ultimately allow to re-identify at least 17% of the US population (about 52 million individuals) (*141*). The results and overall implications of the attack by the Census have been debated. For example, Muralidhar (*142*) argues that the attack yields not only one but many candidate reconstructed datasets, and an adversary would not necessarily be able to determine which one is correct. Francis (*143*) argues that the population-level information released by the Census would allow an adversary to make inferences with better accuracy than what can be achieved by using the Census’s reconstruction attack. The debate was exacerbated by the fact that few technical details were available on the attack until recently (*142*). A group of researchers—including some working for the Census Bureau—recently published a paper (not yet peer-reviewed) describing in detail a second reconstruction attack and its empirical results on 2010 Census data (*140*). The paper acknowledges some criticism of the original attack—specifically on the effectiveness of the attack compared to a population-level inference baseline—but shows that the second attack achieves 95% accuracy on the most vulnerable records, much higher than the relevant statistical baselines. To our knowledge, these findings have not been disputed.

Overall, while the theory of reconstruction attacks is well established, research on their applicability and effectiveness in practice is relatively scarce. Because of the theoretical “power of linear reconstruction attacks” (*106*), it is possible that future work will show that some widely available summary statistics are considerably more vulnerable to re-identification than previously expected, even under realistic adversary models. We consider this line of investigation as one of the most interesting and impactful ones in the field of anonymization.

### Machine learning models

ML algorithms are increasingly adopted by organizations to automate tasks and to inform or improve decision-making. Examples of ML applications include language translation, voice detection, content moderation, medical diagnosis, and drug discovery. The datasets used to train ML models are often personal data, consisting of records that encode information about individuals, such as demographics, behavioral traces, text messages, or images. The resulting trained ML models may or may not be anonymous, depending on their vulnerability to privacy attacks (*11*). Privacy attacks against ML models are a very active area of research, focusing primarily on MIAs (*144*–*151*) but with some works also investigating attribute inference attacks (*152*–*154*) and reconstruction attacks (*146*, *155*, *156*). These attacks have studied adversaries with different auxiliary information and levels of access to the model: from black-box query access—giving the adversary access to model predictions, as is typical in the context of MLaaS—to white-box access—additionally giving the adversary access to the model’s internal parameters, as is typical in the context of on-device deployment.

Shokri *et al.* (*144*) proposed the first MIA against ML models. Their seminal work demonstrated how models trained via popular MLaaS platforms (developed by Google and Amazon) without any defenses can leak with high accuracy the membership of records. The attack quantifies the membership leakage of classification models via their black-box confidence scores, where each score is a proxy for the probability that the record belongs to the corresponding class. Intuitively, models tend to be more confident on their training records compared to unseen records. The methodology is based on training the same model on similar datasets with and without the record of interest before training a meta-classifier to predict whether the record was part of the training dataset or not. Known as the shadow modeling technique, this generic approach was first developed by Ateniese *et al.* (*157*) and is the main building block of most inference attacks against ML models. This attack was later extended to other setups, including models that only output the prediction label instead of detailed confidence scores for all the classes (*150*) and models released as a white-box (*147*, *148*).

The accuracy of an MIA is generally affected by the size of the training dataset, the level of overfitting (i.e., the gap between the model accuracy on training and on unseen records from the same distribution), the model architecture, and the number of classes. In seminal work, Yeom *et al.* (*145*) showed formally that the level of overfitting provides a lower bound on MIA vulnerability. For instance, models of the same architecture are generally less vulnerable when they are less overfitted (*144*). However, for a similar level of overfitting, some architectures leak more membership information than others, with recent results suggesting that more accurate models may be more vulnerable (*151*). In some cases, even simple classifiers such as logistic regression or decision trees may leak membership of their records, particularly on high-dimensional datasets with a large number of classes (*158*). The vulnerability generally decreases with the training dataset size and increases with the number of classes (*144*). Finally, the accuracy of the adversary’s auxiliary information on the target record also affects MIA performance, with noisier records lowering the effectiveness of the attack (*158*). In general, expressive models—such as overparameterized neural networks—may memorize the presence of at least a few records, particularly rare or atypical ones. Recent theoretical work suggests that some amount of memorization may be needed to achieve optimal model utility on long-tailed distributions (*159*). Empirically, the risk can be higher for records belonging to small subpopulations (*160*) or outlier records (*149*). The common metric used to quantify MIA vulnerability—the average success rate—may not directly reveal such cases, as an attack may perform poorly on the average record but still very confidently infer the membership of some records. Recent work has argued that MIAs should be considered a risk even when they confidently target only a few records (*149*, *151*). We refer the reader to the recent work by Dionysiou and Athanasopoulos (*161*) for a detailed overview of the settings where MIAs are more likely to succeed.

Black-box MIAs have received great attention for two main reasons: First, they assume a weak threat model that applies to the MLaaS setting (*144*, *147*, *161*); second, they can be used as a simple test of the privacy offered by a model. When successful, they can point to leakages in the training pipeline. The standard attack methodology, however, relies on the rather strong assumption that the adversary has access to many records of the underlying data distribution (used to train the shadow models) and has knowledge of the training algorithm and some of the hyperparameters used. Although these assumptions might be strong compared to what would be realistic in most settings, leakages detected in this way should not be dismissed. In practice, when releasing a model, it is important to evaluate on a case-by-case basis whether the hypotheses underlying the attacks are met. The few works that examined more realistic adversaries suggest that the performance of MIAs can be affected when relaxing some of the aforementioned assumptions (*144*, *146*, *151*, *161*).

Attribute inference attacks were first studied by Fredrikson *et al.* (*152*) in the black-box setting, and later improved by Mehnaz *et al.* (*153*) and extended to the white-box setting (*154*, *162*). The study by Fredrikson *et al.* was based on a real-world use case, asking whether a model to predict a patient’s dosage of the warfarin drug can be exploited to learn a patient’s genetic marker. McSherry (*163*) called into question the conclusions of this study, arguing that since genetic markers are correlated with the dosage and the adversary’s previous knowledge includes the dosage, it is unclear whether the model leaks more individual-level information than what can be inferred from the previous knowledge. A similar argument was put forward recently by Jayaraman and Evans (*154*), who evaluated the performance of various attribute inference attacks against baseline approaches that perform imputation based on previous knowledge available on the distribution. Their results suggest that, at least in the settings they consider, current black-box attacks may not be able to extract more information about the target than the imputation baseline, while white-box attacks might. They also highlight the need to distinguish, when evaluating attacks, between inferences made possible through access to a model and population-level inferences that are possible even without access to the model.

Reconstruction attacks aim to recover one or more records of the training dataset given access to the model. Intuitively, generative models are more directly exposed to reconstruction attacks, as their goal is to produce outputs similar to the training dataset. Language models for text generation have been shown to output sensitive text sequences from their training datasets, including personally identifiable information (PII) such as names or social security numbers (*156*, *164*). In the context of classification models, reconstruction attacks have so far only been demonstrated in constrained setups. In 2015, Fredrikson *et al.* (*162*) proposed techniques for extracting a representative sample of a class. Balle *et al.* (*155*) later showed that a “worst-case” adversary—one that has complete knowledge of the dataset except for one record and white-box access to the model—can reconstruct the unknown record from the model parameters. While such a strong adversary model is useful to measure worst-case information leakage, it is unclear how these findings translate to privacy risks in practice.

Defending against privacy attacks on ML models is presently a very active area of study. Training models with differential privacy guarantees is regarded as the most robust and principled way to prevent attacks, although current techniques often lead to a high cost in utility (or at the cost of weak formal privacy guarantees). We refer the reader to the “A Theory for Releasing Aggregate Data: Differential Privacy” section for an overview.

### Synthetic data

While data query systems and precomputed statistics can provide better privacy protection than de-identification, this can come at a cost in usability: Analysts need to adapt the statistical tasks they wish to perform to the interface provided by the data query system or to the statistics that have been published. SDG is one of the approaches that aim to circumvent this issue, by having the released aggregates take the form of an artificial dataset of the same format as the original data (*165*).

Two main flavors of SDG exist, which aim to protect against different threat models: partially and fully synthetic data. Partially synthetic data, introduced by Little (*166*) and Reiter (*167*), aim to protect against inference attacks for a specific set of sensitive attributes that is defined by the curator. This is done by replacing their values with values obtained by an ML model trained to infer them from supposedly nonsensitive attributes. Hence, the partially synthetic dataset consists of the original individual-level records, with the original nonsensitive attributes and “artificial” sensitive ones. The rationale is the same as de-identification techniques based on quasi-identifiers, such as *k*-anonymity: an adversary knowing the nonsensitive attributes of a target user should not be able to learn their sensitive attributes. Partial SDG does not perform aggregation on the full records. It is rather a form of record-level de-identification, as part of the individual-level records is preserved and can be used for linkage attacks. Therefore, it suffers from the same limitations as other de-identification methods, in particular regarding the arbitrariness of what is considered to be nonsensitive and the difficulty to apply them to high-dimensional dataset (see the “Record-Level Data” section). In the rest of this section, we use the expression synthetic data to refer to fully synthetic data.

Fully synthetic data generation (*168*, *169*) means that the records shared with the analyst are fully sampled only from aggregates, and specifically from a data generation model. The original data are only used for the purpose of training an SDG model, producing the aggregates, e.g., the weights of the model, and are never released (*168*). Any record is thus fully synthetic, “breaking the link” between the generated records and real persons. Different approaches have been proposed to generate synthetic data. These include explicit models such as Gaussian, vine copulas (*170*), graphical models (*171*) [in particular acyclic dependency graphs (*172*) and Bayesian networks (*173*)], agent-based models (*174*), and implicit models such as generative adversarial networks (GANs) (*175*). Since 2014, the introduction of GANs has rekindled research interest in high-dimensional generative models. GANs have been used to synthetize electronic health records (*176*), mobility trajectories (*177*), and even human faces (*178*). A few general-purpose tools for SDG mechanism have been released, such as the Synthetic Data Vault (*170*) and synthpop (*179*).

As there are no links between synthetic records and real persons, (fully) synthetic data prevent the linkage attacks used against de-identified datasets. However, as for aggregate statistics and ML models, this is not always enough to guarantee privacy: The generated dataset can still leak information about individuals in the training dataset. Several attacks have been proposed, which assume access only to the synthetic dataset, not to the trained model—a setup known as the no-box attack setting (*180*). For instance, the synthetic data might contain records that are statistically “close” to real records with higher probability when these records are in the real data, hence allowing simple distance-based MIAs (*181*, *182*).

Recent works have studied the privacy risks of synthetic data in the no-box setting beyond distance-based attacks. Stadler *et al.* (*183*) propose both membership and attribute inference attacks against SDG, showing that outlier records can be vulnerable, including when using some SDG methods that were supposed to be differentially private. Their MIA uses a shadow modeling technique similar to the one used by Pyrgelis *et al.* (*133*) on aggregate location data. This MIA is, however, mostly ineffective against arbitrary records, while results for their attribute inference attack are given only for outliers. Annamalai *et al.* (*109*) show that attacks on synthetic data can be effective beyond outliers, and show that this is the result of individual-level information leakage rather than population-level inference. Their attack is based on linear reconstruction and relies on substantial auxiliary information, but they show that some attacks can achieve good accuracy against arbitrary records while relying on weaker adversary models.

White-box attacks against SDG, including explicitly exploiting access to the underlying generator and discriminator of a GAN, have also been proposed for MIAs (*184*). Their results show how access to the parameters of the trained model can in some cases substantially increase vulnerability to membership inference, as the adversary can exploit properties of the model that may not be leaked when only the synthetic records are released (e.g., trained parameters of the generator). To facilitate the privacy evaluation of generators under different threat models, Houssiau *et al.* (*180*) have recently proposed an adversarial framework generalizing attacks from previous work.

Synthetic data have recently attracted considerable interest from practitioners (*185*), as they give analysts access to (artificial) individual-level records, allowing them to use the same techniques and pipelines as before, at least in the data exploration phase. From a utility perspective, it is important to remember that, most of the time, there are no guarantees that results of analysis performed on synthetic data will hold true when applied on the real data. Other anonymization techniques not based on ML may, in principle, have the same problem, but they often allow for more control on utility, for example, by knowing exactly the distribution of the noise that is added to the statistics. Synthetic data have so far shown mixed results in terms of utility. For example, Annamalai *et al.* (*109*) assess the utility of synthetic data in terms of statistical error when computing three-way marginal queries. They find that nondifferentially private SDG does not achieve a good privacy-utility trade-off, but observe that, in some cases, recent differentially private methods can provide both good privacy and reasonable utility (albeit using parameters that give weak theoretical guarantees; see the “A Theory for Releasing Aggregate Data: Differential Privacy” section). Moreover, they show that, for a fixed trained model, both privacy and utility of synthetic data heavily depend on the number of synthetic records that are generated. On the other hand, Stadler *et al.* (*186*) study the utility (and privacy) of synthetic data for different use cases, such as the training of ML and the analysis of outliers. They find that the privacy-utility trade-off is no better than the one achieved by some traditional record-level techniques that include generalization and *k*-anonymity.

Overall, we thus see synthetic data as a very useful tool for testing new systems and for exploratory analysis, but its accuracy strongly depends on the use case and any findings may need to be validated on the real data. To our knowledge, there have been few large-scale releases of synthetic datasets serving as an anonymized version of personal data. Among the main ones, England’s National Health Service published a synthetic dataset generated from A&E records (*187*) using a Bayesian network. The International Organization for Migration, in collaboration with Microsoft, has released several synthetic datasets to counter human trafficking (*188*), with the latest ones using differential privacy (*189*, *190*). Recently, Israel’s Ministry of Health released a differentially private synthetic dataset of life births, produced in collaboration with researchers of the Boston University (*191*). Some stakeholders eventually asked to implement additional utility and privacy constraints—such as ensuring that no synthetic record is unique—although the researchers were skeptical of their usefulness (*191*, *192*).

## A THEORY FOR RELEASING AGGREGATE DATA: DIFFERENTIAL PRIVACY

Overall, the literature on attacks shows that even aggregate data can, in some cases, be vulnerable to privacy attacks. This prompts the question: Is it possible to provably protect against all re-identification attacks “by default,” including those that have not yet been invented? In the past 15 years, differential privacy has become an extremely popular framework within research to address this question rigorously. While it has proven very useful to reason about privacy leakages in worst-case scenarios, its application in practice has been more challenging. To our knowledge, most implementations have used differential privacy in ways that cannot guarantee that no attack is possible, at least in theory. This does not mean that such implementations are vulnerable in practice: A growing body of research suggests that differential privacy may provide a good privacy-utility trade-off even in the absence of (meaningful) formal guarantees.

### Differential privacy: Definition, properties, and main mechanisms

Proposed by Dwork *et al.* in 2006 (*193*), differential privacy is a formal definition of privacy that can be applied to any mechanism that processes personal data. Intuitively, for a mechanism that takes datasets as input, differential privacy requires that the (probability distribution of the) mechanism’s output does not depend much on the data of any single individual in the dataset. In turn, this guarantees that releasing the output of the mechanism leaks little information about the individual contributions to the final aggregated output. A core intuition behind differential privacy is that privacy is better quantified (and easier to work with formally) as a property of the mechanism—e.g., an algorithm executed by a data query system—not of its output (contrary to, e.g., *k*-anonymity). The key property that ensures privacy is randomization: No deterministic algorithm can satisfy differential privacy. Differential privacy allows the data curator to study and limit the worst-case “information leakage” regardless of the adversary’s capabilities, including the auxiliary information available to them.

The worst-case “information leakage” is controlled by a parameter ε ≥ 0, called the privacy loss (or privacy budget). The higher the ε, the lower the privacy protection. Formally, a randomized mechanism *M* is ε-differentially private if

$$P\left[M\left(D\right)\in S\right]\leq e^{\varepsilon }\;P\left[M\left(D\prime \right)\in S\right]$$

for any set of outputs *S* and for any two neighboring datasets *D*, *D*′. In the original formulation of differential privacy, two datasets are neighboring if they differ only by one record (i.e., all the data about one user). This definition, known as user-level differential privacy, corresponds to the intuitive protections outlined above, which guarantee that the full information about each individual is “hidden.” As we discuss below, this definition can be relaxed in several ways to achieve better utility at the cost of weaker privacy guarantees. Unless stated otherwise, when we speak of differential privacy, we always refer to the user-level definition.

Consistently with the scope of this review, we consider differential privacy only in the centralized model—also known as central differential privacy—where there is a curator that holds the original (personal) data and seeks to release anonymous data. We note that differential privacy can be adapted to other settings, most importantly the local model. Local differential privacy is a variation of differential privacy where the data are randomized on the user’s device before being sent to the curator (*30*). Unlike differential privacy for the centralized model, local differential privacy guarantees (if implemented strictly) that even the curator cannot learn any sensitive information about the user.

The main advantage of differential privacy is that it provides provable guarantees of privacy that are context-independent and completely determined by the value of ε. These guarantees can be translated into a worst-case measure of robustness against attacks, for example, in terms of Bayesian inference (*194*) or hypothesis testing (*195*). Moreover, contrary to de-identification privacy models, the guarantees are independent of the auxiliary information available to any adversary. If ε is small, differential privacy protects any possible victim—no matter how vulnerable—from any privacy attack that exploits the target user’s record. Dwork and Smith (*196*) argue that the choice of ε is essentially a social question, but safe values should not exceed ln 3 (≈ 1.10). Intuitively, this means that any attack exploiting the target user’s record can work only up to exp[ln(3)] = 3 times better than if the record was not included in the dataset at all.

Importantly, ε is only a theoretical, worst-case measure of the effectiveness of privacy attacks. First, it is possible that an ε-differentially private mechanism *M* is later found to be ε′-differentially private, with ε′ < ε [see, e.g., Abadi *et al.* (*197*)]. Second, even if ε is the minimum value such that *M* is ε-differentially private, this does not mean that ε is a meaningful quantification of the privacy risk in practice. The guarantees of differential privacy hold even against adversaries that have perfect knowledge of the full original dataset except for one bit—a much stronger adversary model than those required for most practical contexts. In other words, it is possible that no realistic privacy attack can achieve the level of effectiveness that would, in principle, be allowed by ε. As we later discuss, considerations on practical privacy risks are common in real-world applications of differential privacy, as limiting the theoretical privacy loss to small values while preserving utility is often challenging.

Differential privacy has three fundamental properties. First, it gives a measurable bound for the total privacy loss over multiple differentially private data releases (a property called composability). Specifically, the total privacy loss ε is bounded by the sum of the loss of each release—although, as explained, in many cases it is possible to obtain tighter bounds, which is the aim of much of the research on differential privacy (*30*). Second, differential privacy is closed under post-processing: If the output of an ε-differentially private mechanism is processed by another function (without accessing the original data), the overall mechanism remains ε-differentially private (*30*). Third, the protection offered by differential privacy extends to groups of individuals, with the protections (as quantified by ε) decreasing smoothly (specifically: linearly) with the size of the group (*30*). These three properties make differential privacy a “well-behaved” notion that can be studied in a principled way using formal tools. In particular, composability allows the data curator to easily assess the total worst-case leakage from multiple data releases—a property that is very useful in practice. Although these properties may sound natural, they are not satisfied by most earlier definitions of privacy. For example, *k*-anonymity does not satisfy composability: The combination of two *k*-anonymous datasets may not be *k*-anonymous (*20*). These properties, along with the strong guarantees of differential privacy, are among the main reasons researchers have adopted it as a de facto standard for privacy.

In practice, differential privacy is generally achieved by adding random noise to the original query outputs. The simplest method is the Laplace mechanism (*193*): For a given function *f*, the mechanism evaluates *f* on the input dataset and adds to the result a random noise value drawn from a centered Laplace distribution. To ensure that every individual is protected, the standard deviation of the Laplace distribution must be scaled proportionally to the inverse of the chosen ε multiplied by the sensitivity of the function *f* being released. The sensitivity is defined as the maximum change that adding or removing any user from any dataset can have on that function. Not all differential privacy mechanisms are based on noise addition, however. For example, McSherry and Talwar (*198*) proposed the exponential mechanism, which can be applied to release both numerical and nonnumerical data (e.g., categories, strings, and trees). The mechanism requires the curator to define a score function describing the “utility” of each possible output for each input dataset. At runtime, the mechanism randomly samples an output with a probability proportional to the score for the given dataset. While the Laplace and the exponential mechanisms are general and simple to use, their straightforward application often results in low utility (*199*) or may not be computationally feasible (*198*). Instead, both mechanisms are typically used as building blocks for more complex and effective mechanisms.

### Differential privacy in practice

The privacy-utility trade-off achieved by differential privacy heavily depends on the use case and mechanism. Differential privacy works well to release statistics that have low sensitivity, i.e., functions for which removing or adding one user’s record does not change the output much (*200*). A typical example are counts, i.e., statistics on the number of users that satisfy a certain property (for example, being female under 40). This is very useful, as many data analysis tasks can be performed using only collections of counts (*30*, *201*), such as histograms, marginals, and contingency tables. Releasing many counts is, however, challenging. For example, releasing a full contingency table involves computing a number of counts that grow exponentially with the dimension of the dataset (i.e., the number of attributes). Another important example is time series, such as location data: Because the number of counts grows rapidly with time, releasing aggregate time series is challenging while ensuring user-level differential privacy (*134*, *202*, *203*).

Another practical challenge is how to set the sensitivity—or, more precisely, how to adapt the function to obtain a lower sensitivity. Since differential privacy must “hide” the contribution of any user, it can be difficult to release statistics with high sensitivity, i.e., where a user’s contribution can potentially have a large impact on the result (e.g., with data following long-tailed distributions). Ad hoc strategies to overcome this have been proposed. For example, a possible solution for highly sensitive functions is to remove the outliers or truncate the values within a certain interval (*204*). These measures may, however, introduce bias for commonly used statistical estimators, which must be taken into account to preserve the truthfulness of the analyses (*205*). Researchers have proposed more advanced techniques to address high sensitivity, such as requesting the analyst to make a “guess” on the range of the function on the given dataset and adapt the mechanism’s response to that guess (*200*), or automatically replacing the worst-case sensitivity value with a carefully crafted alternative that depends on the given dataset (*206*). These methods, however, require manual intervention and do not always guarantee good utility (*30*). A more effective approach is often to consider if the same use case can be carried out using different functions that have lower sensitivity, such as robust statistics (*196*).

A general solution to improve the utility of differential privacy is to use more data. It is well known that the number of users in the original dataset affects the privacy-utility trade-off similarly to the privacy loss ε (*30*, *207*). For example, if data about more users are used, it is possible to achieve the same utility while guaranteeing a lower privacy loss ε to all users—a property called scale-epsilon exchangeability, formalized by Hay *et al.* (*208*). Intuitively, this is because the noise added by differential privacy does not depend on the number of users, but only on the sensitivity and on ε. When more users are in the dataset, the “signal-to-noise ratio” for the desired statistic (e.g., the fraction of users satisfying a certain property) improves. While this is a common trend in statistics (e.g., with sampling error) and anonymization, its prevalence in differential privacy can be observed (and formally studied) in mostly any application. This is both a strength and a weakness: Collecting more data is an effective strategy to improve utility in a predictable way, but it also means that differential privacy can be less practical to analyze data that refers to a small number of individuals, e.g., to study the incidence of rare diseases. Similarly, differential privacy can have a disproportionate impact on accuracy for different groups, with lower utility retained on underrepresented populations (*209*).

While datasets relating to few users constitute a challenge for any anonymization technique, the formal nature of differential privacy allows for little flexibility even for use cases where that might be appropriate. This is because the guarantee of a differentially private mechanism must hold independently of the context. On the other hand, in practice, even a heavily vulnerable mechanism (such as releasing exact counts; see the “Attacks on Aggregate Data” section) might be enough to produce reasonably anonymous data in some cases. For example, results of local elections are publicly available as exact counts. This makes them theoretically vulnerable to attacks—for instance, if a candidate receives zero votes, this reveals that their partner did not vote for them. Yet, few would argue that they should be treated as personal data under the GDPR, which would introduce many data protection obligations that would be widely seen as unnecessary and disproportionate in this case. In some cases, exact counts can be formally shown to satisfy a meaningful notion of privacy. For example, Desfontaines *et al.* (*210*) show that exact counts satisfy a variation of differential privacy that can take into account the adversary’s knowledge.

The previous example illustrates another fundamental aspect of the work on differential privacy, where privacy is mostly seen as a property of the mechanism, not of the dataset or, even less, of the context. Such a context-independent approach has several important advantages. For example, if a mechanism is provably privacy-preserving, its privacy properties hold automatically in any context where it is used. Moreover, mechanisms are generally much more tractable with formal methods than datasets or, even worse, real-world contexts. However, this is at odds with the legal definitions and practice of anonymization, where anonymity is a property of the data (see, e.g., Recital 26 of the GDPR) and where the context often plays an important role in determining if data are anonymous (*10*, *211*, *212*).

This difference of approaches can be seen even in work that expressly seeks to reduce the gap between the legal and technical perspectives on privacy. For instance, Cohen and Nissim (*213*) propose predicate singling out, a formal notion of privacy breach that aims to capture the GDPR concept of “singling out”. Intuitively, a mechanism is vulnerable to predicate singling out if an adversary can find a predicate that is true for exactly one user in the original dataset, with probability larger than an appropriate baseline. Cohen and Nissim show that differential privacy prevents singling out, while *k*-anonymity does not. Although these findings are a profound demonstration that *k*-anonymity does not inherently prevent singling out, some of their results rely on assumptions that may not hold in all contexts. For example, the adversary is assumed to have complete knowledge of the underlying distribution from which records are sampled. Moreover, a successful predicate might be “unnatural” and not necessarily correspond to a meaningful violation of anonymity in some contexts. As such, predicate singling out may be very useful to assess whether a certain mechanism can qualify as a “universal anonymizer” under the GDPR, but determining whether a user can be singled out in a given dataset in a specific context would likely require (also) a contextual assessment [as repeatedly indicated by courts; see, e.g., *Breyer* v. *Bundesrepublik Deutschland* (*212*) and Dautlich *et al.* (*211*) for other examples].

All these challenges and limits highlight a simple yet important fact: Differential privacy is an important framework to address and reason about privacy risks, but is not a “magic” solution to every anonymization problem. In particular, differential privacy cannot overcome the Fundamental Law of Information Recovery. Releasing too much information will eventually either leak individual-level information (if the total privacy loss is not bounded) or require too much noise and hence destroy utility (if the total privacy loss is bounded). Researchers have long studied differential privacy from a theoretical perspective, aiming at understanding what tasks can and cannot be performed with strong guarantees. This theoretical line of research has proved important facts on the limits of differential privacy, including on the number of counting queries that can be released [see, e.g., Ullman (*214*) and Dwork *et al.* (*131*)] and on the utility that can be achieved for time series (depending on the size of the original dataset) (*202*). A survey by Vadhan (*207*) provides a detailed overview of these results.

Such impossibility results often apply not only to differential privacy but also to any mechanism that protects against certain privacy attacks (*110*), reflecting the fundamental nature of the privacy-utility trade-off. For example, the issues arising from high sensitivity and a large number of queries are not exclusive to differential privacy, but rather constitute a general problem for anonymization. In many cases, it can be proved that differential privacy is “optimal,” in the sense that it achieves the best possible utility that can be achieved without compromising privacy—according to specific definitions of privacy, utility, and optimality [see, e.g., Vadhan (*207*)]. Most of these results are, however, asymptotic, which makes them not directly applicable in practice, especially for smaller datasets. Moreover, the specific notions of privacy and utility used in optimality results may not capture all the requirements of the specific use case. As explained, data and context are relevant factors to determine if a certain notion of privacy is appropriate. The same goes for utility. For example, Kulynych *et al.* (*215*) show that differential privacy can exacerbate predictive multiplicity, i.e., the phenomenon by which different classification models (trained with differential privacy) achieve similar accuracy, but produce conflicting predictions on the same input. Predictive multiplicity can be seen as a type of arbitrariness and, depending on the setting, can be a cause of concern (*215*, *216*).

### Differentially private ML and SDG

Despite the challenges, the promise of differential privacy—ensuring privacy by setting a small privacy loss ε, without the need to assess the vulnerability to attacks—is an appealing one, especially for types of data where risks are not yet well understood. Also for this reason, the application of differential privacy to ML and to SDG has attracted a growing interest among researchers and businesses.

Training models with differential privacy guarantees is currently seen as the most principled way to prevent privacy attacks, although how to achieve comparable utility (accuracy) to nonprivate models (i.e., models produced without privacy guarantees) with low ε remains an open problem. Early work (*217*) extended the output perturbation approach of Dwork *et al.* (*193*) to various classification models. The most popular approach today is to introduce noise during the training, through objective perturbation by adding noise to the loss function (*217*, *218*), or through gradient perturbation (*197*, *219*). The former is typically used to train relatively simple models such as logistic regression, while the latter is used for deep learning models, which require many passes over the dataset during training. This is challenging with differential privacy, as each access to the dataset increases the total privacy budget used. A key innovation to enable gradient perturbation methods with small values of the privacy budget has been the development of improved composition bounds and methods to track the privacy budget over training iterations (“privacy accounting”) (*197*). One such method, the moments accountant, is one of the key features of DP-SGD, a popular training algorithm implemented in the main deep learning libraries, including PyTorch through the Opacus API (*220*) and TensorFlow (*221*). Other defenses include the private aggregation of teacher ensembles (PATE) (*222*) that also provides differential privacy guarantees.

The main limitation of differentially private ML today is that using low values for the privacy loss ε that give strong privacy guarantees often results in much lower utility compared to the nonprivate models (*209*, *223*). Efficiently adding noise tailored to each model is difficult (*224*). Properly integrating differential privacy in the model development process further involves privacy-preserving hyperparameter tuning (*225*). For domains where a lot of data are easily available, using a larger private training dataset, a similar public dataset for training feature extractors, or robust handcrafted features can improve utility (*226*). Some architecture choices that have become dominant based on their strong performance in the nonprivate setup may achieve sub-optimal utility when trained using DP-SGD, with recent work arguing for the development of privacy-aware architectures (*227*). For instance, Papernot *et al.* (*227*) show that models using a bounded nonlinearity function such as the hyperbolic tangent reach much better utility than the standard ReLU.

Fortunately, empirical evidence suggests that even large values of ε might be sufficient to prevent some attacks (*148*, *164*). This potentially points to a gap between the theoretical privacy afforded by a mechanism against the strongest possible adversary assumed by differential privacy and its robustness to weaker, practical adversaries. This gap can be addressed by formally analyzing the privacy loss under weaker adversary assumptions, but this is considered difficult in nonconvex models (*228*). A practical alternative is to develop auditing approaches (*228*, *229*), where well-defined adversaries (that are weaker than the “worst-case” one implicitly assumed by differential privacy) are deployed against a mechanism, and the attack performance is used to empirically derive a lower bound of the privacy loss. Put informally, a model that leaks this amount of individual-level information must have been trained with a larger ε than the estimated lower bound.

Similarly to ML models, research is ongoing to evaluate whether noise can be added to SDG models to enforce differential privacy at a reasonable cost to utility (*171*, *173*, *230*–*232*). In most cases, this is accomplished with standard techniques from the differential privacy literature, either through noise addition on aggregate statistics (*171*, *173*, *230*) or techniques from the ML literature to train generative networks (*231*, *232*). Although research on differentially private SDG is fairly new, it is growing and increasingly attracting attention. For example, the US National Institute for Standards and Technology (NIST) recently organized a challenge to develop SDG models for several public use datasets with differential privacy (*233*). As mentioned in the “Attacks on Aggregate Data” section, the International Organization for Migration and Israel’s Ministry of Health have both published differentially private datasets. Both use cases use a large theoretical privacy loss: ε = 12 and ε = 9.98, respectively (*191*, *234*).

### Relaxations of differential privacy

To deal with the difficulties of enforcing the standard definition of differential privacy, relaxations have been proposed, which aim to allow for better utility, generally at the cost of weaker—at least in theory—privacy guarantees (*82*, *235*). A commonly used relaxation is event-level differential privacy (*202*), particularly for set-valued datasets where records contain several “events,” e.g., location traces with many data points representing time and location of places visited by the user. In event-level differential privacy, the definition of neighboring datasets states that two datasets are neighbors if they differ by one event, rather than one user. For example, with location data, event-level differential privacy would ensure that an adversary would not be able to establish if a user visited a certain place at a certain time but might, in principle, be able to exploit recurrent patterns in the user’s trace to infer their home address with good accuracy (*236*). Hence, contrary to the original (user-level) definition, event-level differential privacy provably protects each user action but does not protect a user’s full record. This means that data produced with event-level differential privacy may—in theory—be vulnerable to strong inference attacks, hence failing to ensure that the data are anonymous.

Another popular relaxation is approximate differential privacy [also known as (ε,δ)-differential privacy], which allows for a slightly larger privacy loss than the original one, with the allowed margin depending on the delta parameter (*237*). An important property of approximate differential privacy is that, unlike the original one, it is satisfied by the addition of Gaussian noise, which has useful statistical properties. For instance, its tails decrease faster, meaning that large deviations are less likely than for the Laplace mechanism. Tolerating a small δ also allows some operations that cannot be performed with pure differential privacy (e.g., approximately counting all unique values that a categorical attribute takes without computing counts for all possible values, which is crucial for attributes that have very large domains), and for improved composition bounds (*30*). For this reason, Gaussian noise has been shown to lead to good results for ML models (*197*). A recent survey by Desfontaines and Pejó (*82*) proposes a taxonomy of about 200 variants of differential privacy across different dimensions, such as the auxiliary information and computational power that the adversary is assumed to have.

### Real-world applications

Differential privacy has become a very active field of research, with thousands of papers published on the subject. Despite this, differential privacy (and its variations) has so far seen only a small number of real-world applications, almost exclusively in the noninteractive setting (*238*). The first real-world implementation was in 2008, when the US Census Bureau deployed a mechanism proposed by Machanavajjhala *et al.* (*239*) to release commuting patterns of the US population. There have then been no reported applications for several years, but new deployments started again in 2016, with their number growing faster in the past few years (*240*), including for some notable data sharing use cases. In a joint initiative with Harvard’s nonprofit Social Science One, Facebook used differential privacy to produce a rich collection of statistics on publicly shared links, with the aim of enabling independent research on the impact that social media have on democracy (*241*). During the COVID-19 outbreak in 2020, Facebook and Google released differentially private aggregate mobility data, which governments and researchers can use to study the population’s response to confinement policies (*242*, *243*). Microsoft published a dataset that estimates the broadband coverage in about 32,000 US zip codes, using user-level differential privacy with ε = 0.2 (*244*). The Wikimedia Foundation uses Tumult Analytics (*245*) to release daily differentially private pageview data for Wikipedia (*246*). This application is particularly interesting as it combines the traditional server-side processing of differential privacy with a client-side filtering mechanism (*247*). In particular, the mechanism runs on the user device without relying on unique persistent identifiers, but allows to bound the total contribution of the user to the pageview data—i.e., the sensitivity of the statistics that are computed by the server (*246*). Since 2020, Google has been using differential privacy (in various forms and in combination with federated learning) (*29*) to train next-word prediction models in the Gboard keyboard app (*248*). Recently, the guarantees have been improved, with ε between 0.994 and 13.69, depending on the language. According to Google, this is the largest known real-world deployment of user-level differential privacy, and the first real-world use case where models are trained directly on user data with ε < 1.

The most important deployment of differential privacy to date has come from the US Census Bureau, which has used it to release many of the public statistics for the 2020 census—a decision motivated by the discovery of reconstruction and re-identification risks in the traditional disclosure limitation techniques used for the 2010 census (see the “Attacks on Aggregate Data” section) and by the technical properties of differential privacy. Among these, an important one is that the guarantees of differential privacy are not affected by transparency. The parameters and the details of the mechanisms can be safely disclosed, allowing anyone to audit the privacy guarantees and enabling analysts to design statistically sound analyses for the published data by taking into account the characteristics of the added noise (*249*). This is in stark contrast to techniques previously used by the Bureau, whose effectiveness relied on the secrecy of the implementation (*249*)—an approach that goes against best practices in security and privacy, which generally reject security through obscurity (*250*). Despite these advantages, the decision to adopt differential privacy sparked a debate, with many analysts raising concerns regarding the accuracy of the data—particularly the alleged presence of statistical bias concerning minorities (*251*)—and others claiming that the impact is very limited (*252*). Moreover, the Bureau announced that, contrary to original plans, they will not use differential privacy for the next American Community Survey (*253*).

The interactive setting has seen even fewer applications of differential privacy, probably because of the difficulty of managing (and limiting) the privacy loss ε. To our knowledge, the only interactive systems have been deployed by LinkedIn (*254*) and Uber (*255*). LinkedIn’s Audience Engagements API is used for marketing analytics, but while it can be used interactively, the type of queries that can be issued is very limited. Uber deployed an implementation of Chorus, a system proposed by Johnson *et al.* (*255*) that automatically modifies SQL queries issued by the analyst, ensuring that the query enforces differential privacy on itself.

The number and characteristics of real-world uses of differential privacy reveal the hard challenges that come with applying it in practice. First, despite some exceptions, most of these use cases adopt a theoretical privacy loss ε between 2—a fairly large value—and 28.8 (*238*)—an extremely large value that makes the theoretical guarantees essentially meaningless. Second, in many cases, only an event-level relaxation of differential privacy is enforced. This means that the “privacy unit” is not the user but rather the user’s action or set of actions within certain time periods (e.g., a day). In some cases, the total theoretical privacy loss for the user is actually unbounded, such as for Google’s COVID-19 Community Mobility Reports, where updated statistics are released periodically (*243*), meaning that the data release could, in principle, be vulnerable to arbitrarily effective attacks.

Whether these practices constitute a practical risk for privacy is an active area of research (*203*), and there is growing empirical evidence that differential privacy might provide good protections even for large values of the total loss ε (*109*, *140*, *148*, *164*, *256*)—making it a very useful anonymization technique when combined with an assessment of the vulnerability to attacks. The problem has also been studied with a formal approach. For example, Ghazi *et al.* (*257*) use per-attribute differential privacy—a variant of differential privacy—to show that a large ε can, in some cases, provide much tighter yet provable guarantees on the privacy of single attributes taken in isolation. However, the guarantees of per-attribute differential privacy are necessarily context-dependent. For example, its guarantees may not be meaningful if the attributes are heavily correlated (in a way that depends at least partially on the individual user—as opposed to population-level correlations—e.g., for visited places in location history data).

Real-world deployments using large ε would therefore always require an assessment of potential attacks and practical risks. In the local setting, Gadotti *et al.* (*256*) recently proposed an attack and found that Apple’s implementation of differential privacy in iOS and macOS devices may reveal a user’s sensitive information, such as their political orientation. Abowd *et al.* (*140*) show that the reconstruction attack they execute on 2010 Census data would not be effective on the differentially private 2020 census data. Yet, they indicate that, due to the large ε, they cannot rule out that any attack is possible. To our knowledge, these are the only two works assessing the vulnerability to practical attacks of a real-world deployment of differential privacy. Although large ε values do not necessarily translate into actual privacy risks, the difficulty of applying differential privacy with small ε raises the question of the usefulness of ε as a worst-case measure. If meaningful worst-case bounds can rarely be met in practice, is the focus on the formal guarantees of differential privacy justified, or even useful?

Finally, we note that turning mathematically defined mechanisms into code may introduce vulnerabilities that can weaken or even nullify the expected guarantees of differential privacy. This is a real issue, as researchers have repeatedly found bugs in implementations of differentially private mechanisms that may be exploited by attacks. Most notably, bugs can underestimate how much noise must be added to enforce differential privacy with a certain ε (*258*) or can leak information due to finite-precision arithmetic in computers (*259*, *260*). Researchers have proposed methods to automatically detect such violations. For example, Bichsel *et al.* (*261*) recently proposed an ML-based algorithm that can work even in a black-box setting, i.e., when the specifications of the mechanism are not known.

Notably, efforts to simplify the use of differential privacy through dedicated software libraries have intensified in the past few years, both within and outside academia. Much work has gone into the development of software such as Google’s Privacy on Beam (*262*), Harvard University’s OpenDP (*263*), and Tumult Analytics (*264*) that simplify both the application of differentially private mechanisms by data holders and their use by analysts. Such developments are very encouraging, as they have the potential to support a much wider adoption of differential privacy in practice, even by organizations that do not have the resources to implement differential privacy from scratch.

However, software libraries alone cannot completely eliminate the difficulty of using a low ε while preserving utility. Although breakthroughs are always possible, it seems unlikely that a large fraction of the real-world uses of data will be able to retain good utility while ensuring a small (theoretical) privacy loss anytime soon. Ad hoc optimizations for the specific dataset and use case can substantially improve the privacy-utility trade-off, but these typically require considerable time and resources. We expect that the number of applications will increase, but in many cases—especially for the interactive setting—the total theoretical privacy loss allowed by the system will be too large to provide any meaningful mathematical guarantee of privacy. Applying differential privacy, even with a large ε, will likely remain the best way to systematize a careful evaluation of the risks and to avoid the most egregious attacks. As long as differential privacy systems will use a large or even infinite theoretical privacy loss ε to ensure good utility, auditing such systems against known and new attacks will be necessary to empirically quantify the privacy risks in the specific context. This is especially relevant when differential privacy is used as an anonymization technique, as regulators are likely to expect an assessment of the robustness against re-identification, particularly when large ε values are used.

## OUTLOOK

For decades, anonymization has been one of the main ways to share data while protecting privacy. The research on anonymization is becoming increasingly sophisticated, with much work being done on advanced attacks and formal guarantees. These results have progressively shown the inadequacy of pseudonymization and de-identification techniques that many had considered sufficient for anonymization. Pseudonymization—the mere removal of direct identifiers—does not offer any protection from simple linkage attacks that exploit the uniqueness of the records. Record-level de-identification—including popular techniques such as *k*-anonymity—presents inherent vulnerabilities, and there is no reason to believe that it will ever provide an acceptable privacy-utility trade-off for modern, high-dimensional data. The weaknesses of pseudonymization and record-level de-identification have been established beyond reasonable doubt because of a wide range of attacks that can be carried out by a realistic adversary. Overall, some limited auxiliary information about the target user is often sufficient to confidently identify their records and learn sensitive information about them with high accuracy.

The picture for aggregate data is more nuanced. While there is considerable evidence—including theoretical results—that individual-level information can in theory leak from any type of aggregate data (as long as enough information is released), few works have studied whether and how attacks could be carried out by a realistic adversary. In the noninteractive setting, most proposed attacks have so far assumed a strong adversary having access to large amounts of auxiliary information about users other than the target user. Moreover, successful attacks against summary statistics and ML models have so far been mostly demonstrated for membership inference. Despite it being a violation of anonymity, there are use cases where vulnerability to membership inference may be deemed tolerable in practice. This is especially the case if carrying out a successful attack would entail efforts and risks—including legal ones—that appear disproportionate for the expected gains, i.e., the amount and accuracy of the inferred information. Nevertheless, successful membership inference may indicate that the data are vulnerable to stronger attacks, including reconstruction attacks. Moreover, new attacks are of course likely to improve on existing ones, in terms of both effectiveness and adversary models. As such, reasonable safety margins should be used to ensure that the anonymization process is future-proof.

By contrast, attacks against interactive data query systems have been shown to be very effective even with limited auxiliary information, with attribute inference and even reconstruction attacks being realistic threats if the system provides enough flexibility to the analyst. On the other hand, the interactive nature of data query systems allows data curators to implement additional measures that might mitigate the risk that an adversary can successfully execute attacks. These include, for instance, mandatory authentication for the analysts and keeping a log of all queries issued by any analyst to detect possible attack attempts.

Differential privacy has attracted most efforts from privacy researchers since it was proposed in 2006, and it is seen by many as the most promising solution for robust anonymization. The pledge of differential privacy—i.e., context-independent provable guarantees of anonymity that hold against present and future attacks—is an attractive one for researchers, policy-makers, and practitioners alike. However, its adoption in practice has been more challenging than expected. The number of real-world applications of differential privacy is currently limited, and in most cases, the total theoretical privacy loss ε does not provide meaningful formal guarantees of privacy. Despite this, the number of applications is growing fast, with some providing user-level guarantees with small ε. Moreover, there is encouraging evidence that differential privacy often protects privacy even at larger ε values, but such an assessment must necessarily rely on attacks, which generally depend on the context.

Overall, in the research on both attacks and defenses for aggregate data, there is a gap between what is possible in theory and what has been shown to work in practice. To ensure that attacks are useful tools to measure actual risk and are legally relevant, we believe that researchers should focus more on realistic adversary models—which reflect the concept of “means reasonably likely to be used” for identification found in the GDPR. In the case of differential privacy, assessing vulnerability to realistic attacks can be a principled way to accelerate its adoption whenever the worst-case privacy loss ε is too large to rule out attacks a priori. At least in the short to medium term, combining empirical research on attacks with formal defenses is likely to be the most practical way to unlock the potential of data while preserving privacy.
